# Bacterial Extracellular Vesicles: New Hype or Hope to Explain Reproductive Host–Microbiota Interactions

**DOI:** 10.1002/jev2.70296

**Published:** 2026-05-15

**Authors:** Hannah Wein, Paula Iglesias‐Moreno, Apostol Apostolov, Andres Salumets, Damián O. Muzzio, Alberto Sola‐Leyva

**Affiliations:** ^1^ Department of Obstetrics and Gynecology University Greifswald Greifswald Germany; ^2^ Division of Obstetrics and Gynecology, Department of Clinical Science Intervention and Technology, Karolinska Institute Huddinge Stockholm Sweden; ^3^ Department of Gynecology and Reproductive Medicine Karolinska University Hospital Huddinge Stockholm Sweden; ^4^ Celvia CC AS Tartu Estonia; ^5^ Department of Biotechnology Institute of Molecular and Cell Biology University of Tartu Tartu Estonia; ^6^ Department of Obstetrics and Gynecology Institute of Clinical Medicine University of Tartu Tartu Estonia

**Keywords:** bacterial extracellular vesicles, fertility, microbiome, postbiotics, pregnancy

## Abstract

Rapid advances in microbiome research are transforming our understanding of human health and disease, with growing focus on the female reproductive tract as a critical but understudied niche. Evidence for a local microbiome largely derives from bacterial nucleic acid detection; however, the biological relevance of these signals remains debated, with whether they reflect viable microbial communities, transient colonisation, or mere microbial remnants with immunomodulatory effects. Bacterial extracellular vesicles (BEVs) have emerged as pivotal mediators of host–microbiota crosstalk. Their small size enables them to traverse tissue barriers, enter systemic circulation and access barrier‐protected anatomical sites like the placenta, thereby extending their biological reach beyond the site of origin. Although BEVs have been extensively characterised in the context of gastrointestinal, respiratory and systemic diseases, their relevance within the reproductive tract remains insufficiently defined. Understanding whether BEVs contribute to processes such as endometrial receptivity, gamete interaction, embryo implantation and immune tolerance in early pregnancy may reveal novel mechanisms of reproductive physiology and pathology. Furthermore, unravelling the role of BEVs could help resolve the ongoing debate regarding the existence of a functional upper reproductive tract (URT) microbiota, reframing it in terms of microbial activity rather than microbial presence alone. This review synthesises the limited but growing body of evidence on BEVs in the reproductive tract, with a particular emphasis on their potential influence on female fertility and early pregnancy outcomes. We also outline the major methodological challenges, including the discrimination of BEVs from host‐derived extracellular vesicles (EVs), the technical limitations of current detection approaches and the risk of contamination in low‐biomass environments that complicate research in this field. Finally, we highlight conceptual frameworks and future research directions needed to establish BEVs as important players in reproductive biology and to harness their diagnostic and therapeutic potential in reproductive medicine.

## 1 Introduction

Tight regulation of the uterine milieu is critical to render the endometrium receptive and to maintain a successful pregnancy (Mor et al. 2017). Historically, the upper reproductive tract (URT) was considered devoid of microbiota, a notion rooted in the longstanding association of microbial presence with infections and diseases. Nowadays, several microbes have been detected in the uterine cavity, where imbalances (dysbiosis) have been associated with negative reproductive outcomes, such as infertility, recurrent embryo implantation failure, early pregnancy loss and preterm birth (Kyono et al. 2018; Peric et al. 2019; Molina et al. 2020; Toson et al. 2022; Odendaal et al. 2024). Microbial composition of the URT varies with age, parity, menstrual cycle and medical interventions (Chen et al. 2017; Carosso et al. 2020; Wang et al. 2021). The lower reproductive tract, including vaginal mucosa, is well‐documented to be dominated by *Lactobacillus* spp., which also represent the most prominent genus in endometrial tissue. Notably, *Lactobacillus*‐dominance in the endometrium has even emerged as a potential biomarker for in vitro fertilisation success (Peric et al. 2019; Franasiak et al. 2016; Hanaoka et al. 2026). In contrast, the presence of other microorganisms like *Gardnerella*, *Sneathia*, *Prevotella* or *Ureaplasma* has been linked to adverse pregnancy outcomes, including recurrent implantation failure and miscarriage (Aagaard et al. 2014; Moreno et al. 2016; Moreno et al. 2022; Lozano et al. 2023; Vomstein et al. 2024). Such dysbiotic shifts further account for 60% of women with unexplained infertility or recurrent miscarriages (Cicinelli et al. 2014; Cicinelli et al. 2018) and are linked to gynaecological disorders, like endometriosis or adenomyosis (Chen et al. 2017; Khan et al. 2016).

The uterine microbiome is estimated to have bacterial loads up to 10^4^‐fold lower than the vagina (Chen et al. 2017). Due to these extremely low abundances and the risk of contamination during sample collection, the detection of an endometrial core microbial composition or even simply microbial evidence above background signal remains challenging (Molina et al. 2020; Toson et al. 2022; Moreno and Simon 2018; Einenkel et al. 2019; Winters et al. 2019). As up to 80% of human‐associated microbes are non‐cultivable (Dethlefsen et al. 2007), most studies of the URT rely on sequencing‐based approaches (Peric et al. 2019). Although bacterial DNA is frequently detected in the uterine cavity, such evidence does not necessarily indicate the presence of viable microorganisms (Einenkel et al. 2019; Sola‐Leyva et al. 2021; Sola‐Leyva et al. 2026). Additional research is warranted to further elucidate the complex mechanisms of interaction between the host and its microbiome.

Microbial communities are increasingly recognised as modulators of host genome activity and the local tissue microenvironment (Belkaid and Hand 2014; Garrett 2015; Thaiss et al. 2016; Goodrich et al. 2017). The dynamic interplay between the host, its microbiome and pathogenic organisms constitutes a complex and multifactorial biological network. One crucial mechanism by which microbes exert their influence is through the secretion of bioactive compounds, including proteins and metabolites (Xie et al. 2023). Among these mediators, bacterial extracellular vesicles (BEVs) have emerged as key players in interkingdom communication, facilitating the transfer of molecular cargo between microbes and host cells (Diaz‐Garrido et al. 2021). Given their nanoscale size and membrane‐enclosed structure, BEVs may traverse biological barriers more efficiently than intact bacterial cells. Recently emerging studies suggest that microbiome‐derived BEVs can reach the foetal compartment, where they may influence female reproductive health and embryonic development (Kaisanlahti et al. 2023; Turunen et al. 2023).

In this review, we discuss the role of BEVs in human reproductive physiology, with emphasis on their biogenesis, molecular composition and systemic distribution. We synthesise current evidence on host–BEV interactions in the female reproductive tract, covering effects on fertility and infertility, implications for pregnancy and potential roles in foetal immune priming. We further examine the protective potential of BEVs against sexually transmitted infections (STIs). However, one of the primary purposes of the review is to discuss methodological considerations for BEVs isolation, characterisation and functional testing of BEVs, offering state‐of‐the‐art recommendations. Lastly, we consider translational opportunities by exploring the potential of BEVs as postbiotic agents, highlighting critical knowledge gaps for future research.

## 2 Methodology

A comprehensive literature search was conducted up to 1 February, 2026, to synthesise current evidence on BEVs and their potential roles in the female reproductive tract, fertility and pregnancy, with particular emphasis on the detection of BEVs in humans and in vitro studies. Searches were performed in PubMed (MEDLINE) and Scopus using predefined terms related to BEVs and reproductive outcomes, combined with Boolean operators (Table S1). Conference abstracts, letters, study protocols, non‐English publications and articles without full text were excluded. Two reviewers (H.W. and P.I.M.) independently screened titles and abstracts, followed by full‐text assessment. Discrepancies were resolved by consensus. A total of five studies with BEVs from human samples and 14 in vitro studies investigating the role of BEVs in the context of female reproduction and pregnancy were included, consolidating the findings for this review. Additional studies providing insight into the potential role of BEVs on female reproductive health or pregnancy were discussed along the text.

## 3 BEVs in Human Physiology

### 3.1 Biology of BEVs

Historically, it was believed that only gram‐negative bacteria could produce BEVs (Beveridge 1999). However, BEV production is not limited to gram‐negative bacteria, and gram‐positive species also release BEVs (Dorward and Garon 1990; Brown et al. 2015). Due to fundamental differences in cell wall architecture, the biogenesis and molecular composition of BEVs still vary significantly between gram‐negative and gram‐positive bacteria. Gram‐negative bacteria can give rise to outer‐membrane vesicles (OMVs) and outer–inner membrane vesicles (OIMVs), gram‐positive bacteria mainly produce cytoplasmic membrane vesicles (CMVs) (Toyofuku et al. 2019; Toyofuku et al. 2023). OMVs are secreted via blebbing of the outer membrane, resulting in vesicles containing outer membrane proteins and little to no cytosolic content. OIMVs, in contrast, can additionally carry nucleic acids and other cytosolic components, since weakening of the peptidoglycan layer enables protrusion of the inner membrane and the subsequent release of the vesicles. CMVs are formed by endolysin‐triggered rupture of the peptidoglycan layer and protrusion of the cytoplasmic membrane, referred to as bubbling cell death. These vesicles thus contain cytosolic contents, nucleic acids and inner membrane proteins (Toyofuku et al. 2019; Toyofuku et al. 2023). Resulting BEV sizes range from 20 to 400 nm, with the mean size and cargo composition greatly depending on parental bacteria, culture conditions and biogenesis (Toyofuku et al. 2019; De Langhe et al. 2024). Given that the composition widely differs among BEV subtypes, the detection of BEVs in biological samples remains difficult and universal BEV markers are not available to date (Toyofuku et al. 2023; De Langhe et al. 2024).

BEVs from commensal bacteria have been shown to support physiological homeostasis and protect against infection and disease (Jiang et al. 2024). Notably, BEVs can influence vascularisation, extracellular matrix remodelling, cell migration and immune modulation (Yang et al. 2020; Stanton 2021; Qu et al. 2022), processes that are fundamental to embryo implantation and pregnancy progression. The immunomodulatory potential of BEVs has been extensively studied in vitro and in vivo (Kaparakis‐Liaskos and Ferrero 2015; Zhao and Jones 2023; Peregrino et al. 2024). Although most reports describe pro‐inflammatory effects, certain bacterial species produce BEVs with anti‐inflammatory properties. For instance, *Brucella abortus* BEVs attenuate pro‐inflammatory cytokine expression in THP‐1 cells (Pollak et al. 2012), whereas *Mycobacterium tuberculosis* BEVs suppress CD4^+^ T‐cell activation and proliferation (Athman et al. 2017). *Bacteroides fragilis* releases BEVs that induce tolerogenic dendritic cells, which in turn, promote the development of regulatory T cells (Shen et al. 2012). Similarly, BEVs from *Bacteroides thetaiotaomicron* promote dendritic cell‐mediated immune homeostasis (Durant et al. 2020). By altering immune balance, cell invasion/migration and angiogenesis, BEVs could impact reproductive success (Soares et al. 2014).

Macrophages are key immune regulators in the reproductive tract, contributing not only to pathogen clearance but also to angiogenesis, trophoblast invasion and immune tolerance during early pregnancy (Faas et al. 2014; Renaud and Graham 2008; Ning et al. 2016). BEVs can enter macrophages by different endocytosis pathways modulating macrophage activity with their cargo (Imayoshi et al. 2011). BEVs can further elicit both pro‐ and anti‐inflammatory responses in these cells.

### 3.2 Distribution in the Human Body—Horizontal Transmission

**FIGURE 1:**
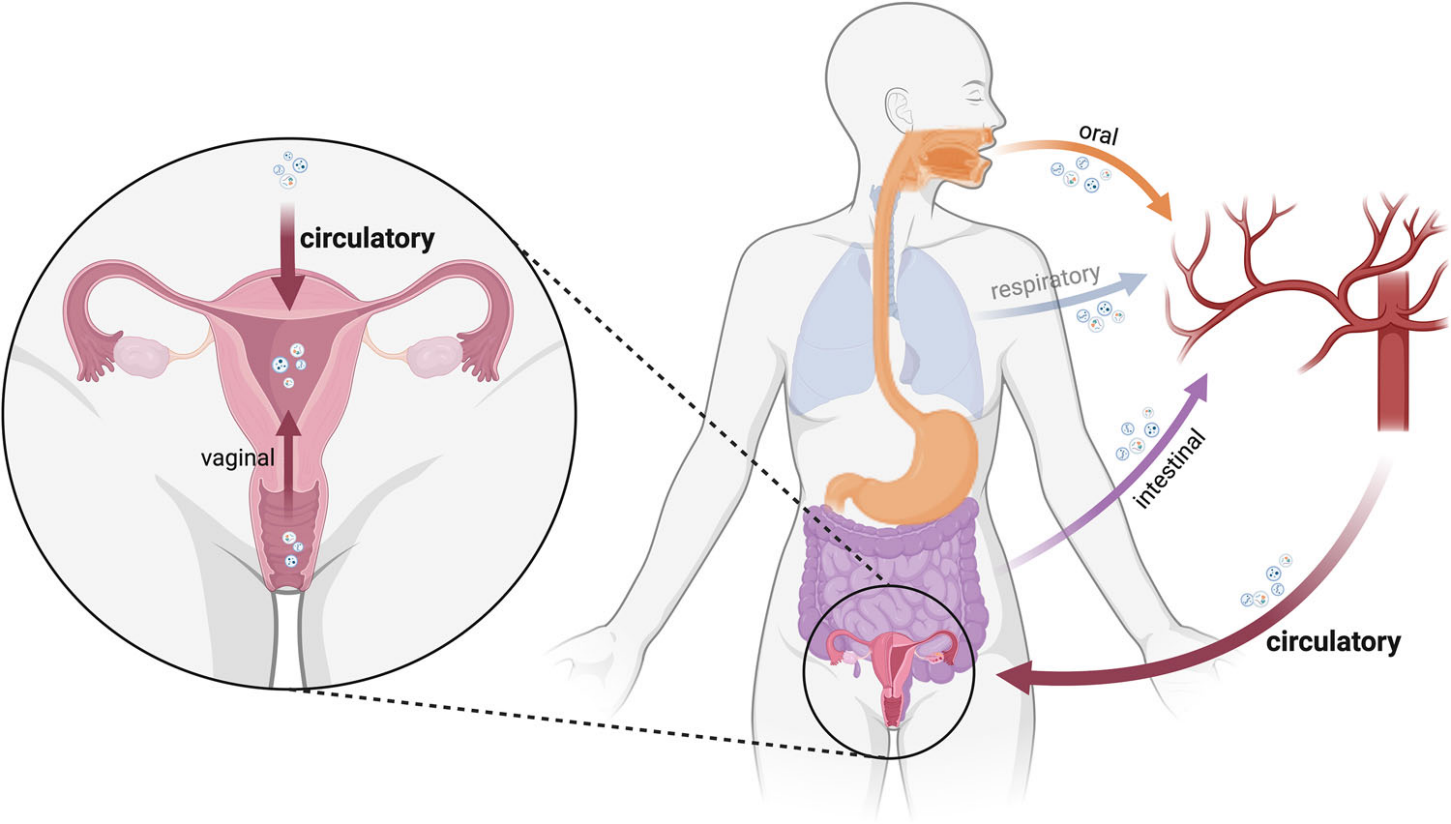
Bacterial extracellular vesicles (BEVs) origin and distribution. The origin of BEVs in the uterus is still to be determined. Although most studies refer to ascension from the vagina, access via circulation might represent an alternative route. Oral, respiratory and intestinal BEVs could surpass tissue barriers entering circulation. The vesicles would then accumulate at sites of high blood throughput, such as the uterus or the placenta (in case of pregnancy). Dysbiotic shifts in the gastrointestinal or oral microflora might consequently affect the uterine milieu, bearing the potential to affect vulnerable reproductive processes. *Created with BioRender.com*.

To exert an influence on local pregnancy‐related processes, BEVs are expected to reach the URT. Growing evidence indicates that BEVs from the vaginal microbiota can ascend through the cervicovaginal mucus (reviewed in Moore et al. 2024; Wang et al. 2021) (Figure 1). Interestingly, several studies have linked shifted oral and gut microbiota with adverse pregnancy outcomes (Amir et al. 2020; Ye and Kapila 2021; Gorczyca et al. 2022; Giannella et al. 2023) such as preeclampsia (PE) (Lv et al. 2019; Chen et al. 2020; Jin et al. 2022; Boggess et al. 2003), gestational diabetes mellitus (Crusell et al. 2018) and spontaneous abortion (Moore et al. 2004; Farrell et al. 2006; Jin et al. 2020; Liu et al. 2021). DNA from oral‐ and gut‐associated bacteria (Eckburg et al. 2005; Tenaillon et al. 2010; Abusleme et al. 2013; Brennan and Garrett 2019; Martinson and Walk 2020), including *Fusobacterium nucleatum* and *Escherichia coli*, has been identified in the healthy placenta (Aagaard et al. 2014; Satokari et al. 2009; Rautava et al. 2012). In fact, comparative analyses revealed that placental microbial DNA exhibits ∼71% similarity to oral microbial communities, suggesting potential hematogenous transfer from the maternal mouth to the placenta (Aagaard et al. 2014; Saadaoui et al. 2025). The nanoscale size and amphiphilic properties of BEVs may further facilitate systemic spread and tissue barrier penetration (Choi et al. 2015; Steinman et al. 2026). In addition to oral commensals, the intestinal phyla *Bifidobacterium* spp. and *Lactobacillus rhamnosus* (Vinderola et al. 2024) were found in the placenta, independent of the mode of delivery (Satokari et al. 2009). The presence of live bacteria in the placenta is strongly challenged (Perez‐Muñoz et al. 2017; Sterpu et al. 2021) and attempts to culture bacteria from placental samples were unsuccessful, suggesting that these signals may represent bacterial remnants rather than an active placental microbiome (Satokari et al. 2009). In a more recent study, BEVs positive for the outer membrane markers outer membrane protein A (OmpA) and lipopolysaccharide (LPS) were successfully isolated from term placenta (Menon et al. 2023). BEV isolates showed near‐complete overlap with 16S rRNA profiles of placental samples, suggesting BEVs as a likely source of microbial DNA rather than live bacteria in the placenta (Menon et al. 2023).

Sequencing analysis of BEVs isolated from human amniotic fluid and maternal faeces, suggested a shared origin of both amniotic fluid‐derived and faecal BEVs in the maternal gut microbiota (Kaisanlahti et al. 2023). The detection of BEVs in the urine of healthy pregnant and non‐pregnant women supports the hypothesis of in vivo circulation of BEVs in women (Yoo et al. 2016) (Figure 1). A promising model for tracking BEV dynamics in vivo is the intravenous and oral application of stained BEVs to mice (Table 1). According to multiple studies in this model, BEVs do, in fact, enter the bloodstream (Park et al. 2017; Stentz et al. 2018) and accumulate within the liver, kidney, colon and partially spleen and lungs (Kaisanlahti et al. 2023; Choi et al. 2015; Jones et al. 2020; Chen, Rao, et al. 2022; Ou et al. 2022). In pregnant mice, orally applied *Akkermansia muciniphila* BEVs were detectable in the placenta and foetus (Chen et al. 2023).

## 4 Host–BEV Responses in Female Reproduction

### 4.1 BEVs and Female Fertility

**FIGURE 2:**
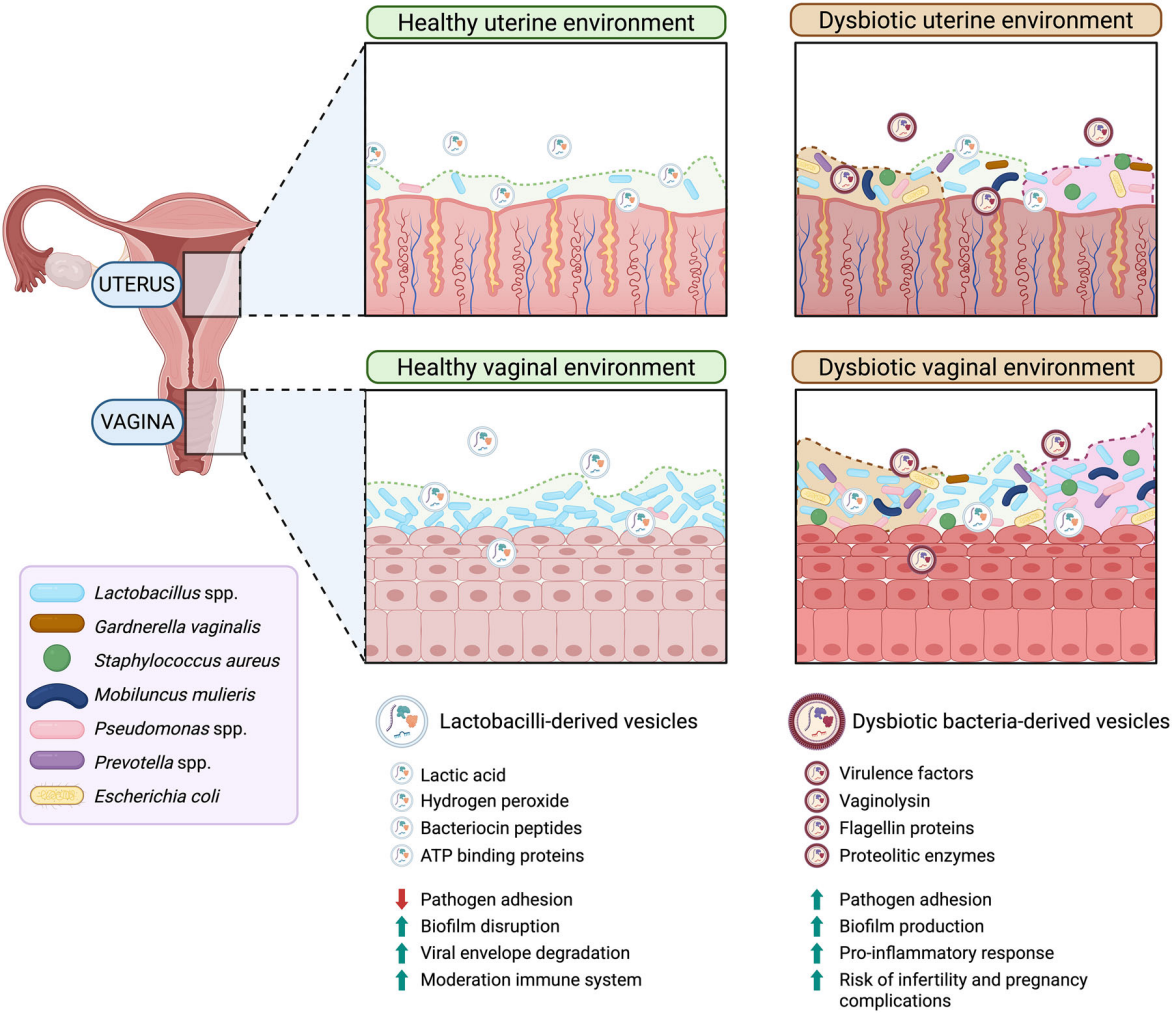
Healthy and dysbiotic microbial environment in the uterus and vagina. The healthy vaginal milieu is *Lactobacillus*‐dominant, while its proportion is considered lower in the uterus. Lactobacilli‐derived extracellular vesicles (BEVs) carry different molecules that promote a healthy vaginal and uterine environment, in contrast, dysbiotic bacteria‐derived BEVs promote biofilm production and disruption of this healthy environment. *Created with BioRender.com*.

**FIGURE 3:**
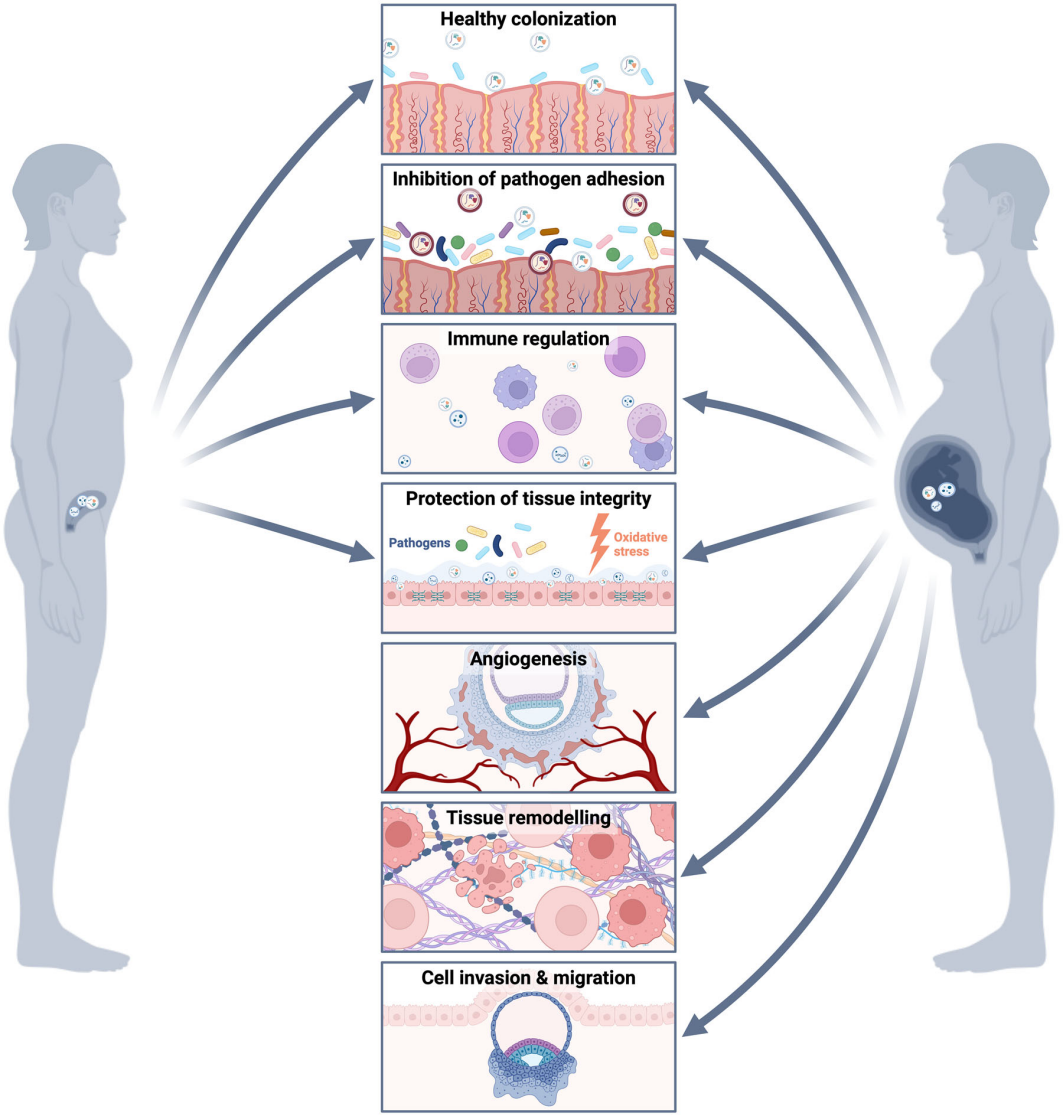
Roles and functions of bacterial extracellular vesicles (BEVs) in healthy woman. BEVs bear the potential to influence the uterine environment with their cargo, promoting healthy microbial colonisation and preventing pathogen growth. Furthermore, effects on immune cell activity and tissue integrity have been proposed. During pregnancy, women might additionally benefit from BEV‐mediated effects on tightly regulated processes, such as angiogenesis, tissue remodelling and trophoblast function. *Created with BioRender.com*.

The female reproductive tract microbiome contributes to local immune balance and tissue health, shaping the conditions necessary for successful pregnancy (Figure 2) (reviewed in Mor et al. 2017; Gao et al. 2024; Wang et al. 2025). To date, few studies have investigated the role of BEVs in female reproductive health. However, the available evidence is largely consistent with findings from studies on live bacteria, underscoring the need for further research (Shishpal et al. 2020; Lee et al. 2021; Croatti et al. 2022; Wang, Lee, et al. 2022; Khan et al. 2023; Artuyants et al. 2024; Joseph et al. 2024) (Table 2). *Lactobacillus* spp. BEVs were shown to reduce opportunistic pathogen adhesion and growth by carrying lactic acid and bacteriocin peptides (Dean et al. 2020) and enhancing the adhesion of lactobacilli to epithelial cells (Croatti et al. 2022) (Figure 3). In fact, in vitro treatment of cervical cancer cells (HeLa) with *Lactobacillus crispatus* BC5 and *Lactobacillus gasseri* BC12‐derived BEVs increased adhesion of lactobacilli up to 335%, while reducing adhesion of opportunistic bacteria, such as *Escherichia coli* and *Staphylococcus aureus* (Croatti et al. 2022). Another study further confirmed the potential of *L. gasseri* BEVs to disrupt vaginal pathogen biofilms (Khan et al. 2023). The species was thus shown to prevent growth of *Lactobacillus delbrueckii* (Croatti et al. 2022), *S. aureus* (Croatti et al. 2022; Khan et al. 2023), *E. coli, Streptococcus agalactiae, Enterococcus faecalis* (Croatti et al. 2022) and *Gardnerella vaginalis* (Khan et al. 2023). *G. vaginalis* releases CMVs with a size range from 120 to 260 nm (Turnbull et al. 2016), which carry a distinct proteomic profile compared to the whole cell lysate or other cellular fractions (Shishpal et al. 2020). Treatment of VK2/E6E7 vaginal epithelial cells with *G. vaginalis* CMVs induced a dose‐dependent pro‐inflammatory response mediated by IL‐8. Moreover, the cell viability decreased significantly upon treatment with concentrations higher than 75 µg/mL CMVs (Shishpal et al. 2020). Interestingly, BEVs from *G. vaginalis* showed altered protein composition and reduced cytotoxicity when treated with lactic acid (Shishpal et al. 2021).

**TABLE 1:** In vivo biodistribution studies on bacterial extracellular vesicles (BEVs) after administration in animal models.

Year	Reference	BEV background	Dye	Animal model	Administration route	Amount applied	Time frame	Organs analysed
2015	Choi et al. 2015	*Phenylobacterium panacis*	Cy7	C57BL/6J mice	Transoral	20 µg	12 h	Liver, heart, lungs, adipose tissue, skeletal muscle
2016	Surve et al. 2016	*Streptococcus agalactiae* A909	FITC	C57BL/6J mice	Vaginal	100 µg BEVs in 100 µL PBS	6 h	Uterus
2020	Jones et al. 2020	*Bacteroides thetaiotaomicron*	DiD	C57BL/6 female mice	Transoral	2 × 10^10^ BEVs in 200 µL PBS	8 h	Gastrointestinal tract, kidney, liver, spleen, heart, lungs
2022	Chen et al. 2022	*Ligilactobacillus animalis*	DiO, DiR	C57BL/6J male mice	Transoral	30 µg in 200 µL PBS	3, 24, 72 h (maximum at 3 h)	Gastrointestinal tract, kidney, liver, spleen, lungs, bones
2022	Ou et al. 2022	*Escherichia coli*	DPASP	Healthy nude mice	Intravenous	∼10^9^ BEVs/mL PBS	0.5, 4, 12, 24 h (maximum at 12 h)	Colon, kidney, liver
2023	Chen et al. 2023	*Akkermansia muciniphila*	PKH26	C57BL/6J pregnant mice (gestational age: embryonic day 17.5)	Transoral	20 µg in 200 µL PBS	5 h	Placenta, foetus
2023	Kaisanlahti et al. 2023	Isolated from faecal samples of pregnant women	Texas red	FVB/NRj pregnant mice (gestational age: end of first trimester)	Intravenous	∼10^8^ BEVs in 100 µL PBS	24 h	Liver, heart, lungs, brain, foetuses

**TABLE 2:** Studies investigating bacterial extracellular vesicles (BEVs) in the female reproductive health.

Year	Reference	BEV background	Bacterial source	Growth phase for BEV isolation	BEV isolation method	BEV characterisation method	Compliance with MISEV guidelines (use of orthogonal methods)	In vitro model	Key findings
Studies on BEVs isolated from human samples									
2016	Yoo et al. 2016	Not applicable	Urine	Not applicable	Differential centrifugation, Ultracentrifugation	NGS, PCR	No	None	Taxonomic BEV composition in the urine differed among pregnant and non‐pregnant women
2021	Lee et al. 2021	Not applicable	Peritoneal fluid	Not applicable	Ultracentrifugation	NTA, Pyrosequencing, Metagenomics	No	None	BEV composition in PF differed among women with endometriosis and healthy controls
2023	Kaisanlahti et al. 2023	Not applicable	Amniotic fluid and maternal faecal samples	Not applicable	Ultracentrifugation, DGC, SEC	TEM, NTA, MS, SDS‐PAGE, PCR, 16S rRNA sequencing	Yes	None	‐ BEVs were detected in human amniotic fluid and maternal faeces ‐ Faeces‐derived BEVs were detectable in the foetus when injected to the tail vein of pregnant mice
2023	Menon et al. 2023	Not applicable	Placenta from caesarean delivery	Not applicable	DGC, SEC	TEM, ExoView analysis, NTA, Western Blot (OmpA, LPS, ALIX), sequencing	Yes	BeWo (ATCC) THP‐1 (ATCC) hFM‐DEC	‐ BEVs were successfully isolated from human term placentae ‐ BEVs further entered both placental and decidual cells and induced pro‐inflammatory cytokine production in THP‐1 monocytes
2025	Dietz‐Ziegler et al. 2025	Not applicable	Faecal samples from pregnant women	Not applicable	Different methods tested, including ultracentrifugation, precipitation	TEM, NTA, MS proteomics, PCR, 16S rRNA sequencing	Yes	Human CD3^+^ T cells	‐ Bacterial origin of faecal BEVs differs between pregnant and non‐pregnant women ‐ Faecal BEVs isolated from faecal samples of pregnant women favour T cell differentiation towards the T_H_2, and reduced differentiation of the T_H_17 phenotype compared to non‐pregnant control BEVs
Studies on BEVs isolated from bacterial cultures and used in in vitro models									
2016	Surve et al. 2016	*Streptococcus agalactiae* (A909, NEM316, 2603 V/R)	University of Washington	Culture to OD_600_ = 1.2	Ultracentrifugation	DNA quantification, TEM, SEM, AFM, DLS, SDS‐PAGE, zymography, PCR	Yes	HeLa	‐ *Streptococcus agalactiae* EVs were internalised by HeLa cells and reduced their viability ‐ BEV treatment induced mechanical weakening and collagen degradation in mouse chorio‐decidual membranes ‐ BEVs translocated to the uterus upon vaginal application in mice ‐ Intra‐amniotic BEV injection triggered inflammation, apoptosis, foetal death, and preterm delivery in mice
2019	Ñahui Palomino et al. 2019	*Lactobacillus crispatus* (BC3, BC5), *Lactobacillus gasseri* (BC12, BC13)	Vagina (from selective bacteria stocks)	Not mentioned, ON culture (10^8^ CFU/mL)	Ultracentrifugation	NTA, SDS‐PAGE, Western blot, ^1^H NMR spectroscopy, MS	Yes	Cell lines: MT‐4 (NIH AIDS Reagent Program), Jurkat‐tat with HIV‐1 tat protein (NIH AIDS Reagent Program), TZM‐bl reporter cells (NIH AIDS Reagent Program) Ex vivo: human cervico‐vaginal and lymphoid tonsillar tissues	EVs from *Lactobacillus crispatus* BC3 and *Lactobacillus gasseri* BC12 inhibited HIV‐1 infection in cervico‐vaginal and tonsillar tissues by reducing viral attachment and entry
2020	Shishpal et al. 2020	*Gardnerella vaginalis*	ATCC	Not mentioned, 48 h culture	Ultracentrifugation	TEM, DLS, LC‐MS/MS proteomics, SDS‐PAGE, Western Blot	Yes	VK2/E6E7, Fresh erythrocytes	‐ EVs from *Gardnerella vaginalis* entered vaginal epithelial cells, causing a cytotoxic response and an increase in IL‐8 secretion ‐ BEVs induced lysis of erythrocytes
2021	Shishpal et al. 2021	*Gardnerella vaginalis*	ATCC	Not mentioned, 48 h culture	Ultracentrifugation	TEM, DLS, Flow cytometry, LC‐MS/MS proteomics, SDS‐PAGE	Yes	VK2/E6E7	pH stress can affect bacterial cell and BEV morphology: changes in protein composition and cytotoxic potential were observed
2022	Croatti et al. 2022	*Lactobacillus* spp.*, Streptococcus agalactiae* *Escherichia coli*, *Staphylococcus* *aureus*, *Enterococcus faecalis* (BC101)	Vagina ATCC, University of Bologna	Not mentioned, 24 h culture (10^9^ CFU/mL)	Ultracentrifugation	DNA/RNA quantification, NTA, DLS	Yes	HeLa	*Lactobacilli*‐derived EVs promoted adhesion of beneficial bacterial species on HeLa cells, and decreased attachment and growth of opportunistic bacteria
2022	Wang et al. 2022	*Lactobacillus crispatus*	BCRC	Stationary phase	Ultracentrifugation	TEM, NTA	Yes	3A‐sub‐E (BCRC)	*Lactobacillus crispatus* EVs reduced cell senescence in placental cells, and protected from oxidative stress by enhancing Akt phosphorylation
2023	Chen et al. 2023	*Akkermansia muciniphila*	ATCC	Not mentioned	Ultracentrifugation	TEM, NTA, Western Blot (OmpA, LPS)	Yes	HTR‐8/SVneo (ATCC)	‐ Oral administration of *Akkermansia muciniphila* EVs mitigated PE symptoms in a mouse model ‐ Orally applied BEVs translocated to the foetus and entered the placenta ‐ BEVs were internalised by HTR‐8/SVneo trophoblasts and significantly elevated their viability and migration capacity through the EGFR‐PI3K‐AKT pathway
2023	Khan et al. 2023	*Lactobacillus gasseri*	ATCC	Not mentioned, 48 h culture	Ultracentrifugation	TEM, DLS, SDS‐PAGE	Yes	*Gardnerella vaginalis* (ATCC) and *Staphylococcus aureus* (ATCC) biofilms	*Lactobacillus gasseri* EVs disrupted the integrity of *Gardnerella vaginalis* and *Staphylococcus aureus* biofilms
2023	Lara et al. 2023	*Porphyromonas gingivalis*	ATCC	Early stationary phase	Ultracentrifugation	TEM, DLS	Yes	HTR‐8/SVneo	‐ *Porphyromonas gingivalis* EVs were internalised by trophoblast cells via clathrin‐ and caveolin‐mediated endocytosis ‐ Internalisation was associated with metabolic arrest, and decreased trophoblast migration and invasion ‐ *Porphyromonas gingivalis* EV injection at the onset of placentation led to reduced placental and birth weight in mice
2023	Lara et al. 2023	*Porphyromonas gingivalis*	ATCC	Early stationary phase	Ultracentrifugation	DNA/RNA quantification, TEM, DLS	Yes	HTR‐8/SVneo, EA.hy926, THP‐1 (ATCC)	‐ *Porphyromonas gingivalis* EVs were internalised by trophoblast cells, where they reduced oxidative stress responses and cell marker expression ‐ Conditioned media from BEV‐treated trophoblasts induced neutrophil recruitment and activation ‐ Conditioned media reduced endothelial cell migration, and promoted monocyte‐endothelial adhesion
2024	Artuyants et al. 2024	*Lactobacillus gasseri*, *Gardnerella vaginalis*	ATCC	ON culture diluted to OD_600_ = 0.05	Ultracentrifugation, DGC, SEC	TEM, NTA, SWATH‐MS proteomics	Yes	Ect1/E6E7 (ATCC) *Trichomonas vaginalis* (B7RC2)	*Lactobacillus gasseri* EVs inhibited adhesion and growth of *Trichomonas vaginalis*, while *Gardnerella vaginalis* EVs contributed to its pathogenicity
2024	Joseph et al. 2024	*Gardnerella vaginalis, Mobiluncus mulieris, Lactobacillus crispatus*	ATCC	Not mentioned, ON culture	Ultracentrifugation	TEM, ZetaView/NTA, MS	Yes	Ect1/E6E7 (ATCC), End1/E6E7 (ATCC), VK2/E6E7 (ATCC), THP‐1, TLR2 (NF‐κB‐SEAP/KI‐IL‐8 Lucia) (InvivoGen)	‐ EVs from *Gardnerella vaginalis* and *Mobiluncus mulieris* were internalised in cervical and vaginal epithelial cells, inducing a multi‐cytokine response via TLR2 pathways ‐ EVs from *Lactobacillus crispatus* were internalised but did not induce inflammatory responses
2025	Hasegawa et al. 2025	*Mobiluncus mulieris*, *Lactobacillus crispatus*	ATCC	Not mentioned, 5 days (*Mobiluncus mulieris*) or 2 days (*Lactobacillus crispatus*) culture, respectively	Ultracentrifugation	ZetaView/NTA	No	Ect1/E6E7 (ATCC), End1/E6E7 (ATCC), VK2/E6E7 (ATCC), THP‐1 (InvivoGen), THP1‐Dua KO‐TLR2 (InvivoGen)	‐ Exposure of cervicovaginal epithelial cells to live *Mobiluncus mulieris*, bacterial supernatants, and BEVs induced transcriptional alterations ‐ BEVs induced the strongest effect, modulating inflammatory cytokine expression pathways via TLR2‐ and TLR5 ‐ BEVs strongly upregulated matrix metalloproteinase expression ‐ Increased *Mobiluncus mulieris* abundance was associated with significantly higher MMP‐9 protein levels from vaginal swabs
2026	Steinman et al. 2026	*Lactobacillus crispatus*, *Lactobacillus iners*, *Gardnerella vaginalis*, *Mobiluncus mulieris*	ATCC	Not mentioned, 3 days culture	Ultracentrifugation	TEM, NTA	Yes	VK2/E6E7 (ATCC), BeWo b30 (ATCC), Ishikawa (MilliporeSigma)	‐ EVs from all 4 bacteria species translocated through cervical mucus (with higher mobility compared to whole bacteria) ‐ BEVs were internalised in vaginal epithelial, endometrial and trophoblast cells ‐ BEVs induced different cytokine responses in these cells, depending on the bacterial background

Abbreviations: AFM, atomic force microscopy; CFU, colony‐forming unit; DGC, density gradient centrifugation; DLS, dynamic light scattering; LPS, lipopolysaccharide; MS, mass spectrometry; NGS, next generation sequencing; NTA, nanoparticle tracking analysis; OD_600_, optical density (at 600 nm); OmpA, outer membrane protein A; ON, overnight; PE, preeclampsia; PF, peritoneal fluid; SEC, size exclusion chromatography; SEM, scanning electron microscopy; TEM, transmission electron microscopy.

Even though there are only few in vitro studies using BEV isolates (Table 2), BEVs might also greatly contribute to the effects observed in studies with bacteria‐free supernatants. In in vitro studies, microbial supernatant from *M. mulieris* disrupted the cervical epithelial barrier through IL‐6 and IL‐8 secretion and miRNA‐mediated mechanisms found in the context of spontaneous preterm birth and shorter gestational time in humans (Dude et al. 2020). Treatment of ectocervical and endocervical cells with *G. vaginalis* or *Lactobacillus iners* supernatants increased cell permeability and cytokine production, while altering the miRNA profile. Interestingly, *L. crispatus* supernatant mitigated these effects (Anton et al. 2018). Analogously, cervicovaginal epithelial cells treated with live *G. vaginalis* or their bacteria‐free supernatant expressed higher levels of MMP‐9, a matrix metalloproteinase involved in tissue remodelling (Anton et al. 2022). Although both live bacteria and supernatants induced weakening of the barrier via MMP‐9, the effect was stronger for live bacteria. Moreover, *G. vaginalis* induced cervicovaginal epithelial cell death and increased the expression of NF‐κB and proinflammatory cytokines upon activation of Toll‐like receptor (TLR) 2 (Anton et al. 2022). *L. crispatus*, both live and supernatant, did not affect cell viability or cytokine secretion but rather reduced MMP‐9 expression (Gerson et al. 2022) (Figure 3).

A more recent study analysed the protein composition of *G. vaginalis* and *M. mulieris* BEVs (Joseph et al. 2024). *G. vaginalis* BEVs contained vaginolysin, a pore‐forming toxin, possibly mediating the observed effects. As *M. mulieris* is a flagellated motile bacterium, its BEVs contain proteins from the flagellin family. BEVs from both opportunistic bacteria induced significant upregulation of several cytokines, thus mediating inflammatory responses, while treatment with *L. crispatus* BEVs did not affect cytokine levels (Joseph et al. 2024). This suggests the involvement of BEVs in the study of cytotoxicity of bacterial supernatants.

### 4.2 BEVs and Female Infertility

The health and function of the female reproductive tract is tightly influenced by its microbiota (Elahi et al. 2025). Alterations in the microbial balance, termed dysbiosis, are characterised by the loss of *Lactobacillus* dominance and are associated with changes in the vaginal microenvironment (Rizzo et al. 2021), including increased proinflammatory cytokine levels and altered immune pathway activity, overall increased the risk of acquiring STIs or experiencing pre‐term birth (Anahtar et al. 2015; Svare et al. 2006). *G. vaginalis* and *M. mulieris*‐derived BEVs thus increased proinflammatory cytokine response in the vaginal epithelium (Steinman et al. 2026). *M. mulieris*‐derived BEVs further increased expression of MMPs, thus contributing to a higher preterm birth risk (Hasegawa et al. 2025) (Table 2).

Imbalances of the vaginal microbiome have also been associated with common gynaecological disorders that affect fertility, such as endometriosis (Moreno and Franasiak 2017; Chadchan et al. 2023) and polycystic ovary syndrome (PCOS) (Sola‐Leyva et al. 2023). Endometriosis is an inflammatory condition characterised by the growth of endometrial‐like tissue outside of the uterus (Zondervan et al. 2018; Saunders and Horne 2021). It affects 6%–10% of women in their reproductive age worldwide (Moradi et al. 2021) and is associated with infertility and pain in the pelvic area (Zondervan et al. 2018). However, the origin and causes of endometriosis remain unclear (Fazleabas 2024). Being a chronic inflammatory disease, endometriosis is suggested to be partly influenced by an imbalance of microbial communities in the reproductive tract (Lee et al. 2021). With regard to BEVs, a significantly increased fraction of *Acinetobacter*, *Pseudomonas*, *Streptococcus* and *Enhydrobacter* BEVs was captured in the peritoneal fluid of the endometriosis group compared to the control, while *Propionibacterium*, *Actinomyces* and *Rothia* BEVs were significantly decreased (Lee et al. 2021). These results suggest an alteration of the BEV composition in the peritoneal fluid of women with endometriosis, and therefore microbial community composition (Lee et al. 2021) (Table 2).

The diagnosis of endometriosis is challenging, as ectopic endometrial growth needs to be confirmed by surgery (Berker and Seval 2015). This emphasises the need for non‐invasive biomarkers. A recent publication has compared EV isolates from cervico‐vaginal fluid extracted via cervical brush or vaginal swab from women undergoing diagnostic endometriosis surgery (Paterson et al. 2024). However, only 0.5% of the DNA was from bacterial origin, indicating that either most EVs were host‐ rather than bacteria‐derived, or that most of the BEVs did not carry cytosolic content, such as OMVs from gram‐negative bacteria (Paterson et al. 2024).

In PCOS, gut microbiome‐derived BEVs have been suggested to exert beneficial effects (Yang et al. 2024; Mandelbaum et al. 2023; Taitz et al. 2023; Díez‐Sainz et al. 2022). These include promoting the growth of beneficial bacteria while reducing the presence of pathogenic species in the gut, both resulting in improved glucose and lipid metabolism and reduced inflammation due to bioactive anti‐inflammatory cargos (Yang et al. 2024; Mandelbaum et al. 2023; Taitz et al. 2023; Díez‐Sainz et al. 2022). Since PCOS is oftentimes accompanied by insulin resistance, one study determined the plasma BEV composition before and after metformin treatment, aiming to identify bacterial taxa involved in the diagnosis and treatment of the disease (Hu et al. 2024). After treatment with metformin, glucose levels and insulin resistance index were decreased. Using 16S analysis on plasma, higher taxonomic diversity and a significant increase in *Streptococcus salivarius* was observed in the treatment group, suggesting that treatment had induced modulation of circulating BEVs (Hu et al. 2024).

### 4.3 BEVs in Pregnancy and Potential Vertical Transmission

Throughout all phases of gestation, the interaction between the developing trophoblast and local immune cells is crucial (Gorczyca et al. 2022). Further contributions of a healthy uterine microbiome on uterine health, fertility and pregnancy have been studied thoroughly (Benner et al. 2018). In vitro analysis allowed the determination of mechanisms by which microbiota act, such as the effects on trophoblast cells. For instance, both *L. crispatus* and *F. nucleatum* were shown to promote trophoblast invasion (Heusler et al. 2021; Yoshida et al. 2021), while low‐abundance *F. nucleatum* additionally mediated macrophage homeostasis (Einenkel et al. 2024). BEVs have been proposed as a relevant source of bacterial DNA in the placenta (Menon et al. 2023) and may represent an additional factor involved in the regulation of this controlled environment.

Placenta‐derived BEVs were shown to be internalised by human foetal membrane‐derived decidua parietalis cells (hFM‐DEC) and BeWo trophoblasts (Menon et al. 2023) (Table 2). Upon uptake, BEVs from *L. crispatus*, *L. iners*, *G. vaginalis* and *M. mulieris* were found to alter cytokine secretion of BeWo‐p30 trophoblasts and Ishikawa endometrial cells (Steinman et al. 2026). Studies on HTR‐8/SVneo trophoblasts revealed further modulatory effects (Chen et al. 2023) (Table 2). *A. muciniphila* BEVs were internalised by these cells and significantly increased cell viability and migration by activating the EGFR‐PI3K‐AKT pathway (Chen et al. 2023). In accordance with these results, exposure to *L. crispatus* culture supernatant promoted HTR‐8/SVneo cell invasion in a transwell assay, whereas direct exposure to the bacteria did not affect invasion (Yoshida et al. 2021). In addition to modulating cell motility, *Lactobacillus* BEVs bear the potential to protect tissue integrity during pregnancy (Figure 3). In an in vitro model, *L. crispatus* BEVs were shown to desensitise 3A‐sub‐E human placental cells from oxidative stress (Wang et al. 2022) (Table 2). Treatment with BEVs reduced cell senescence and cell death, while promoting resistance to H_2_O_2_ induction via increasing mitochondrial fusion.

Regarding immune cells, BEVs isolated from faecal samples of pregnant women were recently shown to induce shifts in T cell abundances favouring T helper (Th)2 differentiation and reducing Th17 abundances in vitro (Dietz‐Ziegler et al. 2025). The authors suggested a favourable role in the maintenance of pregnancy. In macrophage‐differentiated THP‐1 cells, in contrast, placenta‐derived BEVs upregulated pro‐inflammatory IL‐1β and IL‐6 secretion, potentially contributing to the proinflammatory phase of implantation (Menon et al. 2023). Interestingly, when hFM‐DEC were treated with small doses of BEVs from the pathogenic *E. coli* O55:K59(B5):H strain, IL‐6 increase was observed as well, whereas high doses became toxic for the cells (Menon et al. 2023). This emphasises the discrimination between pathogenic‐ and commensal‐derived BEVs in the context of supporting healthy pregnancy. Thus, infection‐associated Group B *Streptococcus*‐derived BEVs were also shown to cause preterm birth, chorioamnionitis and foetal death in mice (Surve et al. 2016).

PE, a pregnancy‐specific hypertensive disorder characterised by new‐onset hypertension and multi‐organ dysfunction after mid‐gestation, has been associated with gut dysbiosis, including increased levels of *F. nucleatum* and decreased levels of *A. muciniphila* (Lv et al. 2019; Chen et al. 2020; Jin et al. 2022). In mice, *A. muciniphila* BEVs were able to enter the placenta, and daily oral administration prevented intrauterine growth restriction, lowered blood pressure and alleviated placental dysfunction, overall ameliorating the PE phenotype (Chen et al. 2023). Additionally, expression levels of markers associated with trophoblast invasion and tissue remodelling (N‐cadherin, vimentin and MMP‐9) were increased in the placenta (Chen et al. 2023). In accordance with these results, another study showed the potential of *A. muciniphila* supernatants in promoting macrophage polarisation towards the anti‐inflammatory M2 phenotype in PE rats (Jin et al. 2022).

Notably, in vitro trophoblast proliferation and migration were improved upon treatment with 10 or 20 µg/mL *A. muciniphila* BEVs, but not 50 µg/mL, suggesting a role for controlled low, non‐excessive doses (Chen et al. 2023). It is crucial not only to differentiate between healthy microbiota and dysbiosis, or between commensal and pathogenic species, but also to distinguish varying degrees of bacterial abundance, ranging from moderate to destructive levels (Einenkel et al. 2019). Previous studies had shown that low‐abundance *F. nucleatum* was able to promote in vitro trophoblast function directly and indirectly via macrophage regulation, while infection‐associated high levels induced excessive inflammation and apoptosis of trophoblast cells (Heusler et al. 2021; Einenkel et al. 2024). Additionally, a switch of M0 macrophages towards the M1 phenotype could be observed in mice upon high‐dose *F. nucleatum* treatment, promoting tissue degradation and clinical manifestation of periodontitis (Chen, Sun, et al. 2022). Observations with *A. muciniphila* BEVs in trophoblast cells (Chen et al. 2023) suggest comparable dose–effect relations for BEVs.

Oral cavity infections, particularly periodontitis, are increasingly recognised as systemic inflammatory conditions, which pose a risk of adverse pregnancy outcomes, including preterm birth, foetal growth restriction, PE, spontaneous abortion and low birth weight (Javaid et al. 2025). Among periodontal pathogens, *Porphyromonas gingivalis*, a gram‐negative anaerobic bacterium and key etiologic agent of chronic periodontitis, has emerged as one of the most common and best studied causative agents (Okamura et al. 2021; Reyes et al. 2018). It has been proposed that *P. gingivalis* exerts its detrimental role on pregnancy outcome systemically through the dissemination of *P. gingivalis*‐derived BEVs or EVs from infected macrophages to the foetomaternal interface (Tanai et al. 2025; Tanai and Okamura 2021). In vitro studies showed that *P. gingivalis* BEVs can be internalised by trophoblast cells (Tanai and Okamura 2021; Lara, Loureiro, et al. 2023) which led to reduced invasive potential, metabolic alterations, oxidative stress and the promotion of proinflammatory responses (Lara, loureiro, et al. 2023; Lara, Sassot, et al. 2023). These alterations resulted in the disruption of the interactions of the trophoblast with endothelial cells, evidenced by reduced endothelial cell migration and enhanced adhesion of monocytes. Furthermore, a loss of the anti‐inflammatory characteristics of the trophoblast was observed, accompanied by an increase of proinflammatory markers and improved neutrophil chemoattraction and activation. Finally, using animal models, functional alterations in the placenta and decreased foetal growth in pregnancies of mice receiving *P. gingivalis* BEVs or EVs from *P. gingivalis*‐infected macrophages were observed (Tanai et al. 2025; Lara, loureiro, et al. 2023). Dysbiotic shifts in the oral cavity, gut or vagina may enforce inflammation in the uterine cavity through a pathologic increase and shift of BEV abundances, thus triggering pregnancy complications (Komine‐Aizawa et al. 2019; Wang et al. 2023).

Building on concepts of maternal microbial components reaching reproductive tissues, studies indicate that microbial components from the maternal environment may also reach the foetus already in utero (Satokari et al. 2009; Roduit et al. 2011; Jiménez et al. 2005). Bacteria have successfully been detected in newborn meconium (Kukkonen et al. 2007), where they showed shared microbial profiles with the placenta and amniotic fluid (Kaisanlahti et al. 2023; Collado et al. 2016). Genetically labelled *Enterococcus faecium* was detectable in the amniotic fluid and offspring meconium upon oral inoculation (Jiménez et al. 2005; Jiménez et al. 2008). Thus, bacterial DNA detected in meconium might be traced back to prenatal transfer in addition to perinatal accumulation (Collado et al. 2016; Jiménez et al. 2008; Mshvildadze et al. 2010; Gosalbes et al. 2013).

Foetal exposure to BEVs in utero could make an important impact on early life priming of the naive immune system towards commensal colonisation in the gut. The presence of bacterial DNA in the placenta or amniotic fluid has been linked to modulation of TLR mRNA expression in foetal meconium (Rautava et al. 2012). Notably, the development of intestinal mucosal and secondary lymphoid tissues is dependent on microbial contact via pattern recognition receptors, such as TLRs (Macpherson and Harris 2004; Blümer et al. 2007; Bouskra et al. 2008; Maynard et al. 2012).

### 4.4 BEVs and Their Protective Role in Sexually Transmitted Infections

In the vaginal microbiome, *Lactobacillus* spp. have been shown to produce BEVs that contribute to the maintenance of mucosal homeostasis and protection against pathogens (Croatti et al. 2022) (Table 2). Emerging research suggests that BEVs may play a crucial role in reducing the host's susceptibility to STIs as well. In fact, a protective role of *L. crispatus* BC3 and *L. gasseri* BC12 BEVs has been demonstrated against HIV‐1 infection in the human CD4^+^ T cell lines MT‐4 and Jurkat‐tat in vitro, and human cervicovaginal or lymphoid tonsillar tissues ex vivo (Ñahui Palomino et al. 2019) (Table 2). The inhibitory effects were dose‐dependent and mediated by reduced viral attachment and entry to the cells. In contrast to these results, BEVs from other strains, such as *L. crispatus* BC5 and *L. gasseri* BC13 could not prevent HIV‐1 infection, but still inhibited viral replication in human tissues ex vivo (Ñahui Palomino et al. 2017). Since BEV sizes and amounts were comparable, the inhibitory activity of *L. crispatus* BC3 and *L. gasseri* BC12‐derived BEVs was suggested to be associated with their composition. According to Ñahui Palomino et al. (2019), higher quantities of amino acids and lactic acid would thus be related to their anti‐HIV‐1 activity. Additionally, HIV exploits host endosomal trafficking pathways involved in EV biogenesis, including Rab GTPase‐regulated mechanisms such as Rab27a‐dependent trafficking, which contribute to viral assembly and release. Although mature virions are not packaged within host EVs, HIV‐infected cells release EVs containing viral components such as TAR RNA, viral miRNAs and the Nef protein. These EVs can affect recipient cells by enhancing viral transcription and altering CD4 and MHC‐I trafficking (Kumari and Banerjee 2026).

*L. gasseri* BEVs were shown to contain antimicrobial proteins with the potential to inhibit growth and adhesion of the vaginal parasite *Trichomonas vaginalis*. BEVs from *G. vaginalis*, in contrast, were enriched with virulence factors enhancing *T. vaginalis* pathogenicity (Artuyants et al. 2024). *G. vaginalis* is typically associated with bacterial vaginosis, thus present in dysbiotic environments. These results not only emphasise the potential of commensal‐derived BEVs to prevent infection but also the risk of disease‐associated dysbiosis to pave the way for other pathogens. The role of BEVs in the context of other STIs is still to be elucidated, however. Improving our current knowledge would offer potential for the assisted promotion of URT health using BEV‐based postbiotics (BBPs).

### 4.5 BEV Research—Current Challenges

When interpreting BEV‐related findings, it is essential to consider the methodological variability in both isolation and characterisation procedures. Due to their small size and heterogeneity, the detection and quantification of BEVs remains a significant challenge, particularly in the low concentrations expected in the URT. Flow cytometry, resistive pulse sensing (RPS), nanoparticle tracking analysis (NTA), fluorescence‐based particle counting (membrane dyes) and dynamic light scattering (DLS) are considered current state‐of‐the‐art techniques for quantifying BEVs. However, their results vary depending on the method used, questioning their comparability (De Langhe et al. 2024; Krzyżek et al. 2023). Additional characterisation approaches include imaging‐based particle counting (transmission electron microscopy [TEM] and Cryo‐electron microscopy [Cryo‐EM]), nucleic acid sequencing, polymerase chain reaction (PCR) and protein‐based analyses such as Western blotting or proteomics (Table 3). Given the diverse origins and subtypes of EVs, contextualised interpretation based on a combination of different methods is strongly recommended. Nevertheless, the routine use of such comprehensive characterisation remains limited, mainly due to high costs and restricted access to resources. As a result, approximately 25% of studies on BEVs omit detailed characterisation beyond basic identification of parent organisms (De Langhe et al. 2024; Krzyżek et al. 2023).

**TABLE 3:** Summarised methodological recommendations for bacterial extracellular vesicles (BEVs) (De Langhe et al. 2024; Choi and Lee 2025; Welsh et al. 2024).

Category	Recommendations
Nomenclature	Use bacterial extracellular vesicle (BEV) as the generic term for vesicles released by bacteria. Complement the generic term with operational descriptors (e.g., isolation method, size range and density fraction) when biogenesis cannot be directly demonstrated. Avoid assigning biogenesis‐based terms unless supported by direct experimental evidence.
Sample type and collection	Clearly report the source of BEVs (mono‐ or polymicrobial culture, in vivo/ex vivo, environmental samples). Limit storage before EV separation/concentration, especially for unfiltered samples. When using in vivo or environmental samples, consider potential contamination by host EVs or EVs from non‐target species. Report sample complexity and any enrichment or depletion steps applied before BEV isolation.
Culture conditions	Report bacterial species/strain, growth phase, viability, seeding density and harvest density. Report media composition, supplements, pH, temperature, oxygenation/aeration, culture format (standing, shaking, biofilm, bioreactor, etc.), preparation details, and any physical or chemical stimulants. Report duration of culture/conditioning before harvest. Check for the presence of unintended microbial contaminants when culturing a specific bacterial strain.
Separation/isolation	Report all methodological details of EV separation/concentration. Prefer gentle methods (e.g., filtration, chromatography) over precipitation or ultracentrifugation where possible. If using density gradient ultracentrifugation, determine densities of (B)EV‐rich fractions for each bacterium and growth condition; report fractions clearly. Include procedural controls to assess bacterial depletion and workflow‐induced endotoxin contamination.
Storage	Report storage duration, temperature, buffer composition and number of freeze–thaw cycles. Clearly distinguish storage conditions before and after BEV isolation.
Characterisation	Include core measurements: size distribution, particle number and macromolecular content. Use multiple, complementary methods for biophysical and biochemical characterisation where possible. Use broad markers: LPS for gram‐negative and LTA for gram‐positive EVs, which are well‐characterised and commercially available. Implement additional PAMP characterisation methods where relevant. Acknowledge limitations: specific markers for many species’ EVs or non‐EV materials remain unavailable. Non‐vesicular co‐isolates may include pili, flagella, phage, and protein, lipoprotein or nucleoprotein complexes. Explicitly note that LPS can exist in non‐vesicular form.
Functional assays	Report normalisation methods for BEV input (e.g., protein assay type, particle number or alternative metrics). Justify the chosen normalisation strategy and include appropriate assay controls.
Controls	Include non‐conditioned medium or non‐inoculated medium controls processed identically to BEV samples. Include negative and technical controls appropriate for downstream functional or compositional assays.

Abbreviations: LPS, Lipopolysaccharide; LTA, Lipoteichoic acid; PAMP, Pathogen‐Associated Molecular Pattern.

A large proportion of current BEV research relies on the isolation of vesicles from bacterial culture supernatants where culture conditions are often not mentioned (Table 2). This is particularly critical, as BEV biogenesis and composition are highly sensitive to variables like medium composition, pH, incubation time, temperature and bacterial growth phase for supernatant collection (Toyofuku et al. 2019; De Langhe et al. 2024; Dai et al. 2025; Rodovalho et al. 2023). Incomplete reporting of such parameters poses a major challenge for reproducibility and cross‐study comparability in BEV research and should consequently be avoided. Thus, standardised characterisation and transparent reporting of experimental parameters are crucial to advancing reproducible and interpretable BEV research (Table 3).

## 5 Future Directions

### 5.1 Methodological Consideration: Evaluating the Genetic Background of (B)EVs in Reproduction

One of the longstanding questions in EV biology is whether DNA is encapsulated within the lumen of EVs or merely associated with their surface (Tsering et al. 2024). Current evidence suggests that, in general, DNA is significantly less abundant in EVs compared to RNA and small RNAs. Studies indicate that DNA is predominantly detected on the surface of EVs, while small EVs (40–200 nm) do not carry DNA as cargo (Jeppesen et al. 2019).

Despite the challenges in extracting DNA from BEVs, researchers have developed methods to analyse their nucleic acid content. A recent approach involves isolating RNA, reverse transcribing it into complementary DNA (cDNA), and subsequently performing 16S rRNA library preparation and sequencing (Kaisanlahti et al. 2023). Specifically, targeted cDNA conversion is conducted on the V3–V4 region of the 16S rRNA gene using specific primers, followed by amplification and library preparation for 16S RNA sequencing (Kaisanlahti et al. 2023). This method has been successfully applied to characterise BEVs in various biological fluids, including amniotic fluid, faecal samples and first‐pass meconium from newborns (Kaisanlahti et al. 2023; Turunen et al. 2023). Its application could be extended to the extraction of BEVs from female reproductive fluids, such as uterine fluid, oviductal fluid and follicular fluid, providing valuable insights into microbial BEV contributions within these environments. The exploration of the cargo of BEVs by proteomics stands as a powerful tool for understanding their biological role in human physiology (Weinberger et al. 2025; Asano‐Inami et al. 2025).

Human EVs in uterine fluid (Apostolov et al. 2025) and oviductal fluid (Li et al. 2023) have been of significant interest due to their role in supporting embryo implantation and development. However, while research has extensively explored the beneficial effects of these EVs, no studies have yet determined which portion of these vesicles originate from bacteria. Understanding the contribution of BEVs to reproductive fluids could open new avenues for investigating the microbial influences on fertility and early embryonic development.

### 5.2 Potential of BEVs as Postbiotics Agents

Probiotics are increasingly acknowledged for their benefits in the maintenance of health and the prevention or synergistic treatment of numerous conditions (Weizman et al. 2005; McKean et al. 2017; Hendijani and Akbari 2018; Ikram et al. 2018; Sun et al. 2022; Tegegne and Kebede 2022). Even in the context of pregnancy, beneficial effects have been described for both mother and child (Kalliomäki et al. 2007; Laitinen et al. 2009; Luoto, Laitinen, et al. 2010; Luoto, Kalliomäki, et al. 2010; Elazab et al. 2013). According to the International Scientific Association for Probiotics and Prebiotics (ISAPP), probiotics are “live microorganisms that, when administered in adequate amounts, confer a health benefit on the host” (Hill et al. 2014). The introduction of live bacteria, however, carries risks such as colonisation resistance, unwanted persistence and transfer of antibiotic resistance genes (Imperial and Ibana 2016; Kristensen et al. 2016; Maldonado‐Gómez et al. 2016; Merenstein et al. 2023). In addition, bacterial viability can be compromised by processing, storage and unfavourable gastrointestinal conditions (acids, proteolytic enzymes, immune cells, etc.) (Wang, Chen, et al. 2022). The substitution of probiotics with BEVs may overcome these limitations and offer promising advantages for future applications. Their small size and stability may allow for targeted delivery, systemic distribution and unfolding of local effects. In line with the definition provided by the ISAPP, with postbiotics being “a preparation of inanimate microorganisms and/or their components that confers a health benefit on the host” (Salminen et al. 2021), BEVs can be considered suitable candidates for postbiotic supplementation. The potential of such BBPs in promoting health and mitigating disease has been widely discussed, although not in the context of female reproductive health (Krzyżek et al. 2023; Chen et al. 2024; Xie et al. 2024).

First experimental approaches suggest that BBPs could support key processes in early pregnancy, such as implantation and placental development through pro‐angiogenic and anti‐senescent effects (Wang, Lee, et al. 2022; Wang et al. 2024; Yin et al. 2019). Additionally, BBPs may become relevant in combating pregnancy complications associated with dysbiosis, such as PE. Oral administration of BEVs from *A. muciniphila*, for instance, alleviated preeclamptic symptoms in mice and improved trophoblast function in vitro (Chen et al. 2023). This offers new perspectives, not only for boosting early pregnancy success but also for the intervention in clinically apparent dysbiosis in female health.

The scope of BBPs is further expanded by BEV bioengineering, a relatively new field of research. Recombinant designs enable the selective loading of targeting ligands or therapeutic agents into BEVs, allowing for precise delivery with reduced systemic side effects. Such recombinant BEVs from *B. thetaiotaomicron* have successfully been applied in mice for targeted protein delivery and immunisation, demonstrating safety and efficacy (Carvalho et al. 2019). Placenta‐specific ligands could guide BBPs to the placenta to exert localised effects, which is particularly desirable during the delicate state of pregnancy.

Considering the possibility of BEVs crossing the placenta, effects on the foetus might become relevant for BBP research (Abrahamsson et al. 2015). Early findings from probiotic studies suggest that maternal microbial supplementation can influence foetal gene expression and neonatal microbiota composition (Rautava et al. 2012; Gueimonde et al. 2006). Although comparable studies with BBPs are still lacking, these insights underscore the need to consider foetal outcomes in future BBP applications during pregnancy.

Although these concepts are still in early stages, they highlight the need for further preclinical investigation into the safety, efficacy and specificity of BBPs, particularly in the context of maternal and foetal health.

## 6 Conclusion

BEVs serve as crucial mediators of host–microbiota interactions, influencing both local and systemic processes, including immune modulation and cell function. Given that the overall composition of the human microbiome plays a significant role in shaping reproductive outcomes, investigating BEVs in the context of host‐bacterial communication is essential for elucidating the complex mechanisms underlying female fertility, embryo implantation and successful pregnancy.

Due to their small size, BEVs are not only expected to reach the female URT transcervically but also via systemic spread from distant organs, such as the gut. Once in the URT, BEVs can exert modulatory effects. Different in vitro and in vivo studies have described beneficial roles of commensal‐derived BEVs, including maintenance of tissue integrity, immune modulation and support of early pregnancy processes, such as tissue remodelling, cell invasion, migration and angiogenesis. Furthermore, BEVs have been shown to enhance the colonisation with beneficial microbes while preventing the invasion of pathogenic bacteria and viruses, thereby significantly decreasing the risk of infection. However, the mechanisms underlying those modulatory activities remain largely unknown. Future work should thus investigate the bioactive properties of BEVs by assessing protein and nucleic acid cargoes. Although 2D cell culture models are commonly used to study BEV effects on the female reproductive tract, 3D organoids and assembloids offer a more precise representation of human tissues and should be prioritised in future investigations.

Growing evidence suggests the involvement of BEVs in the development and clinical manifestation of endometriosis, a condition associated with a dysbiotic microbiome and altered BEV compositions in the uterine fluid. Further research is needed to characterise BEV profiles and determine their impact on female reproductive health and fertility‐related conditions.

In summary, BEVs represent an interesting target for the prevention and therapeutical intervention in the context of female reproductive health. However, their investigation in health and disease remains challenging as low abundances complicate their detection and characterisation in vivo. In addition, ethical and technical barriers hinder the collection of clinical samples from healthy individuals and pregnant women.

The field of BEV research in reproduction has only emerged within the past few years. Existing studies have laid the foundation for further exploration, highlighting the vast scope of knowledge yet to be uncovered.

## Author Contributions

**Hannah Wein**: writing – original draft. **Paula Iglesias‐Moreno**: writing – original draft. **Apostol Apostolov**: writing – original draft. **Andres Salumets**: conceptualisation, writing – review and editing. **Damián O. Muzzio**: conceptualisation, supervision, writing – review and editing. **Alberto Sola‐Leyva**: conceptualisation, writing – review and editing, supervision.

## Funding

This work was supported by the Estonian Research Council under Grants PSG1082 and PRG1076; the Swedish Research Council under Grant 2024‐02530; the Novo Nordisk Foundation under Grant NNF24OC0092384; the Horizon Europe grant NESTOR under Grant 101120075; the Estonian Ministry of Education and Research Centres of Excellence grant TK214 name of CoE; HORIZON‐MSCA‐2024‐SE‐01 TULIP Project (Grant agreement ID: 101236395); the German Research Foundation (DFG) under Grant MU 4404/3‐1 and intramural funding from the University Medicine Greifswald.

## Conflicts of Interest

The authors declare no conflicts of interest.

## Supporting information




**Supporting Information**: jev270296‐sup‐0001‐TableS1.docx

## Data Availability

Data sharing not applicable to this article as no datasets were generated or analysed during the current study.
