# Supplementary material for: Bacterial Extracellular Vesicles: New Hype or Hope to Explain Reproductive Host–Microbiota Interactions
Source: J Extracell Vesicles. 2026 May 15;15(5):e70296. doi: 10.1002/jev2.70296 (PMC13178799; doi:10.1002/jev2.70296)
Supplement: Supplementary file 1 — Supporting Information: jev270296‐sup‐0001‐TableS1.docx [file JEV2-15-e70296-s001.docx]

**Table S1.** Search terms used in PubMed and Scopus.

| **PubMed** |
| --- |
| **Exposure**  ("bacterial extracellular vesicles"[Title/Abstract] OR "bacterial extracellular vesicle"[Title/Abstract] OR "bacterial outer membrane vesicles"[Title/Abstract] OR “outer membrane vesicles"[Title/Abstract] OR  "outer-inner membrane vesicles"[Title/Abstract] OR "OIMVs"[Title/Abstract] OR "cytoplasmic membrane vesicles"[Title/Abstract] OR "CMVs"[Title/Abstract] OR "OMVs"[Title/Abstract] OR "BEVs"[Title/Abstract] OR "microbial extracellular vesicles"[Title/Abstract] OR "microbiota-derived extracellular vesicles"[Title/Abstract] OR "bacterial membrane vesicles"[Title/Abstract]) |
| **Outcomes**  ("female reproductive tract"[Title/Abstract] OR "reproductive tract microbiome"[Title/Abstract] OR "female fertility"[Title/Abstract] OR "vagina"[Title/Abstract] OR "endometrium"[Title/Abstract] OR "infertility"[Title/Abstract] OR "placenta"[Title/Abstract] OR "embryo implantation"[Title/Abstract] OR "pregnancy"[Title/Abstract] OR "immune tolerance"[Title/Abstract] |
| **Scopus** |
| (TITLE-ABS-KEY("bacterial extracellular vesicles") OR TITLE-ABS-KEY("bacterial extracellular vesicle") OR TITLE-ABS-KEY("bacterial outer membrane vesicles") OR TITLE-ABS-KEY("outer membrane vesicles") OR TITLE-ABS-KEY("outer-inner membrane vesicles") OR TITLE-ABS-KEY("OIMVs") OR TITLE-ABS-KEY("cytoplasmic membrane vesicles") OR TITLE-ABS-KEY("CMVs") OR TITLE-ABS-KEY("OMVs") OR TITLE-ABS-KEY("BEVs") OR TITLE-ABS-KEY("microbial extracellular vesicles") OR TITLE-ABS-KEY("microbiota-derived extracellular vesicles") OR TITLE-ABS-KEY("bacterial membrane vesicles"))  AND  (TITLE-ABS-KEY("female reproductive tract") OR TITLE-ABS-KEY("reproductive tract microbiome") OR TITLE-ABS-KEY("female fertility") OR TITLE-ABS-KEY("vagina") OR TITLE-ABS-KEY("endometrium") OR TITLE-ABS-KEY("infertility") OR TITLE-ABS-KEY("placenta") OR TITLE-ABS-KEY("embryo implantation") OR TITLE-ABS-KEY("pregnancy") OR TITLE-ABS-KEY("immune tolerance")) |
